# A novel sub-regional radiomics model to predict immunotherapy response in non-small cell lung carcinoma

**DOI:** 10.1186/s12967-024-04904-6

**Published:** 2024-01-22

**Authors:** Jie Peng, Dan Zou, Xudong Zhang, Honglian Ma, Lijie Han, Biao Yao

**Affiliations:** 1 Department of Oncology, The Second Affiliated Hospital, Guizhou Medical University, Kaili, China; 2 Department of Radiation Oncology, The First Affiliated Hospital of Zhengzhou University, Zhengzhou, China; 3 Department of Radiation Oncology, Cancer Hospital of the University of Chinese Academy of Sciences, Hangzhou, China; 4 Department of Hematology, The First Affiliated Hospital of Zhengzhou University, Zhengzhou, China; 5 Department of Oncology, Tongren People’s Hospital, Tongren, China

**Keywords:** Immunotherapy, Sub-regional radiomics, Non-small cell lung carcinoma, Response

## Abstract

**Background:**

Identifying precise biomarkers of immunotherapy response for non-small cell lung carcinoma (NSCLC) before treatment is challenging. This study aimed to construct and investigate the potential performance of a sub-regional radiomics model (SRRM) as a novel tumor biomarker in predicting the response of patients with NSCLC treated with immune checkpoint inhibitors, and test whether its predictive performance is superior to that of conventional radiomics, tumor mutational burden (TMB) score and programmed death ligand-1 (PD-L1) expression.

**Methods:**

We categorized 264 patients from retrospective databases of two centers into training (*n* = 159) and validation (*n* = 105) cohorts. Radiomic features were extracted from three sub-regions of the tumor region of interest using the K-means method. We extracted 1,896 features from each sub-region, resulting in 5688 features per sample. The least absolute shrinkage and selection operator regression method was used to select sub-regional radiomic features. The SRRM was constructed and validated using the support vector machine algorithm. We used next-generation sequencing to classify patients from the two cohorts into high TMB (≥ 10 muts/Mb) and low TMB (< 10 muts/Mb) groups; immunohistochemistry was performed to assess PD-L1 expression in formalin-fixed, paraffin-embedded tumor sections, with high expression defined as ≥ 50% of tumor cells being positive. Associations between the SRRM and progression-free survival (PFS) and variant genes were assessed.

**Results:**

Eleven sub-regional radiomic features were employed to develop the SRRM. The areas under the receiver operating characteristic curve (AUCs) of the proposed SRRM were 0.90 (95% confidence interval [CI] 0.84−0.96) and 0.86 (95% CI 0.76−0.95) in the training and validation cohorts, respectively. The SRRM (low vs. high; cutoff value = 0.936) was significantly associated with PFS in the training (hazard ratio [HR] = 0.35 [0.24−0.50], *P* < 0.001) and validation (HR = 0.42 [0.26−0.67], *P* = 0.001) cohorts. A significant correlation between the SRRM and three variant genes (*H3C4*, *PAX5*, and *EGFR*) was observed. In the validation cohort, the SRRM demonstrated a higher AUC (0.86, *P* < 0.001) than that for PD-L1 expression (0.66, *P* = 0.034) and TMB score (0.54, *P* = 0.552).

**Conclusions:**

The SRRM had better predictive performance and was superior to conventional radiomics, PD-L1 expression, and TMB score. The SRRM effectively stratified the progression-free survival (PFS) risk among patients with NSCLC receiving immunotherapy.

## Key message

- What is already known on this topic: The relationship between sub-regional radiomics and genomic alterations in the context of immunotherapy for lung cancer has not been reported.
- What this study adds: The sub-regional radiomics model (SRRM) showed better performance in predicting immunotherapy response for non-small cell lung carcinoma than conventional radiomics, tumor mutational burden score, and programmed death ligand-1 biomarkers. H3C4 and PAX5 mutations were associated with the immunotherapy-responsive SRRM-low group, while EGFR mutation was significantly associated with SRRM-high group, especially the L858R mutation sub-type. SRRM-low group showed longer progression-free survival than the SRRM-high group in the training and validation cohorts.
- How this study might affect research, practice or policy: This approach is generalizable to any medical imaging analysis, including immunotherapy, offering a novel noninvasive way to tailor cancer treatment.

## Background

Non-small cell lung carcinoma/cancer (NSCLC) accounts for approximately 80% of all lung cancers, most of which are diagnosed as advanced NSCLC [1]. Recently, immune checkpoint inhibitors (ICIs) have revolutionized the treatment of patients with advanced NSCLC. ICIs alone or in combination with chemotherapy are now recognized as first-line therapy for the treatment of NSCLC or as second-line therapy for patients who do not respond to chemotherapy [2]. The KEYNOTE trial, a phase II/III study showed an overall survival benefit with pembrolizumab over docetaxel in patients with advanced NSCLC [3, 4]. Long-term survival benefit was also observed in patients with NSCLS having a programmed death-ligand 1 (PD-L1)-expression of ≥ 50%. However, usually, only a small number of patients respond to treatment with ICIs, and a small subset of patients demonstrate immune hyperprogression [5–7]. Presently, the biomarkers for ICIs, such as expressions of tumor mutational burden (TMB) and PD-L1, do not provide sufficient predictive accuracy in clinical applications [8, 9]. This lack of precision can lead to challenges in the effective implementation of ICI treatment in advanced NSCLC. Therefore, it is essential to explore a novel biomarker that can accurately estimate which patients would respond to ICI therapy before its initiation.

Radiomics are first-order or higher-order measures that capture quantitative information present in the imaging data [10]. They form an active area of computational medical imaging research because of their non-invasiveness and ability to convey important disease information that would otherwise be invisible to human observers [11, 12]. Previous studies have investigated the use of radiomics in NSCLC, including the assessment of the immune-inflammatory status of tumors, which is thought to play a key role in distinguishing potential responders to ICIs from non-responders [13–15]. Conventional radiomics primarily focuses on the characteristics of the entire tumor, with a lack of quantitative analysis on the heterogeneity within the sub-regions of the tumor. In recent years, the study of tumor sub-regional radiomics has emerged as a promising and rapidly advancing field. This includes research progress in areas such as predicting responses to targeted therapy in breast cancer and prognosis prediction of glioma [16, 17]. Meanwhile, a growing number of studies have combined genomics, radiology, proteomics data, and pathology to estimate PD-L1 expression levels, TMB, and tumor microenvironment (TME) or predict the response to immunotherapy and side effects in patients with cancer [18–20]. Recent studies reported that a machine learning analysis of circulating immune cell characteristics or CT images in patients with NSCLC could be used to predict immunotherapy benefits [21, 22]. However, the utility of combining sub-regional radiomics and machine learning for predicting responses to ICIs in advanced lung cancer remains unclear.

Thus, the aim of our study was to construct a sub-regional radiomics model (SRRM) on computed tomography (CT) scans and test whether its predictive performance was superior to that of conventional radiomics, TMB score, and PD-L1 expression in NSCLC before ICI treatment. Additionally, we investigated the associations between the SRRM on one hand and progression-free survival (PFS) and variant genes.

## Methods

### Patients treated with ICIs

#### MIND cohort

This study used database data from 247 patients with lung cancer from the Memorial Sloan Kettering Cancer Center (MSKCC) cohort (https://www.synapse.org/#!Synapse:syn26642505) [23]. The genomics data were download from cbioportal (https://www.cbioportal.org/study/summary?id=lung_msk_mind_2020). All patients received anti-PD-1/PD-L1 treatment; of these, 22 with missing clinical data or CT images were excluded. Consequently, 225 patients were included in this cohort.

#### TCIA cohort

A total of 46 patients, who received at least two cycles of anti-PD-1 treatment, were identified from a retrospective database available at the Cancer Imaging Archive (http://www.cancerimagingarchive.net/). Seven patients without CT images or assessment of treatment response were excluded. Finally, 39 patients were included.

An aggregate of 264 patients, compiled from both the MIND and TCIA cohorts, were randomly divided into two new cohorts as follows: training cohort, *n* = 159 patients; and validation cohort, *n* = 105 patients; in a ratio of 3:2. This study was approved by the institutional review board of the Second Affiliated Hospital of Guizhou Medical University (2023-LUNSHEN-02) and was performed in accordance with the Declaration of Helsinki. The specific use of these open data sets did not involve any personal information.

### Study design

**Fig. 1:**
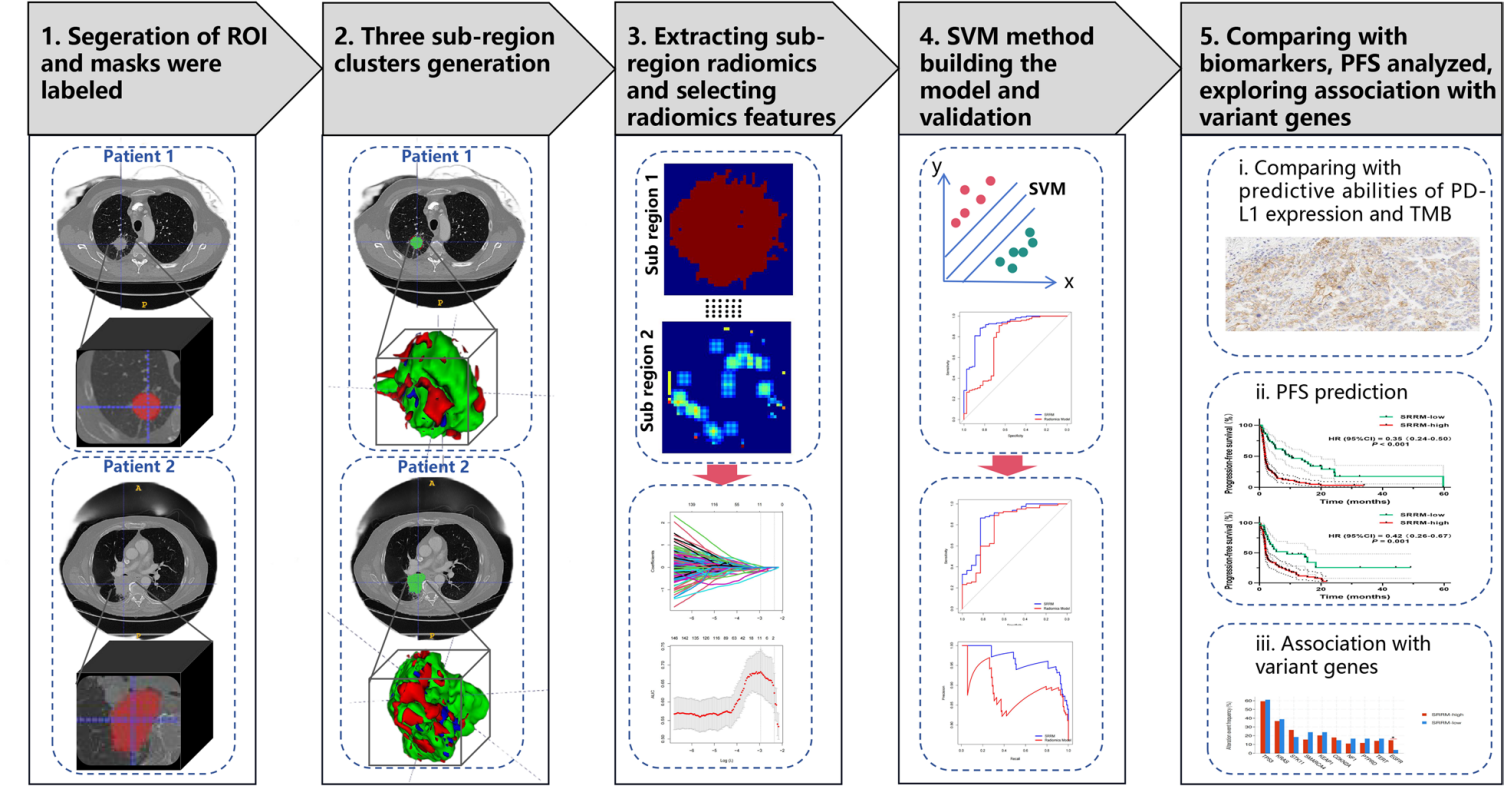
Overview of the workflow in our study. *PD-L1* programmed death-ligand 1, *PFS* progression-free survival, *SVM* support vector machine, *ROI* region of interest, *TMB* tumor mutational burden

An overview of the study design is presented in Fig. 1. The study was conducted as follows: Step 1: We normalized the CT images, and each slice of the tumor area was mask-labeled by ITK-SNAP (http://www.itksnap.org/pmwiki/pmwiki.php) and confirmed by two senior radiologists. Step 2: We applied a K-means clustering algorithm (K = 3) using Python v.3.12 (https://www.python.org/) to divide the tumor into three sub-regions; we did not perform clustering on the conventional radiomics tumor region of interest (K = 1). Step 3: Each sample included three sub-regions, and we extracted 1896 features from each sub-region, resulting in a total of 5688 sub-regional radiomic features for each sample. A total of 1896 radiomics features were also extracted from the whole tumor area by the conventional radiomics method for comparative purposes. After correlation analysis of all features, we removed the redundant features with strong correlation; thereafter, we applied the least absolute shrinkage and selection operator (LASSO) to reduce the dimensionality and preserve the best radiomic features. Step 4: We used the support vector machines (SVM) algorithm to build the model in the training cohort after adjusting the relevant parameters [24], and tested it in the validation cohort. Receiver operating characteristic (ROC) curve analysis of the SRRM was further performed in each of the two cohorts. Step 5: We compared the SRRM with conventional radiomics, TMB score, and PD-L1 expression, and the correlations with PFS and variant genes were analyzed.

### Evaluation of TMB score and PD-L1 expression

All tumor samples from the MIND cohort were analyzed by next-generation sequencing (NGS) before ICI treatment [23]. The test method was performed on the U.S. Food and Drug Administration-licensed Memorial Sloan Kettering Cancer Center’s Integrated Mutation Profiling of Actionable Cancer Targets platform, which includes somatic mutations, copy number alterations, and fusions of 341–468 genes most commonly associated with cancer. Based on NGS profiling in patients from the MIND cohort, we defined a TMB of ≥ 10 mutations (muts)/Mb as “high TMB” and TMB < 10 muts/Mb as “low TMB.” PD-L1 immunohistochemistry was performed on formalin-fixed and paraffin-embedded tumor tissue sections using standard PD-L1 antibody (E1L3N; Cell Signaling Technology, Danvers, Massachusetts, USA). The tumor cells were considered to have a high PD-L1 expression level when ≥ 50% of the cells stained positively.

### Extraction of sub-regional radiomic features

To compensate for differences in radiological characteristics resulting from different reconstruction slice thicknesses and pixel sizes [25], the voxel size of all CT images in this study was reconstructed to 1 × 1 × 5 mm^3^. The volume of interest was normalized to 64 Gy levels to compensate for CT scanner variations. A Gaussian mixture model was used to cluster intratumoral sub-regions with radiomics features. The optimal number of clusters representing the diversity of the tumor ecosystem was identified using the Bayesian information criterion in an unbiased manner, resulting in a cluster of three (K = 3). For each sub-region, region volume, shape, strength, and texture were quantified to 1896 radiomics features using texture analysis and wavelet decomposition methods [26]. For comparative purposes, 1896 radiomic features were also extracted from the whole tumor area and intratumoral sub-regions of each patient. Tumor sub-regional clusters and all radiomics feature extraction were performed in PyRadiomics (version 3.1; https://pyradiomics.readthedocs.io) [27]. All radiomics contain seven feature classes: first order, shape, gray level co-occurrence matrix (GLCM), gray level size zone matrix (GLSZM), gray level run length matrix (GLRLM), neighboring gray tone difference matrix (NGTDM), and gray level dependence matrix (GLDM) features.

### Optimal feature selection, SRRM construction, and validation

To reduce redundant radiomic features, sub-regional radiomic features with high correlations (CC > 0.75) were excluded. The LASSO, utilizing fivefold cross-validation, was applied to select features that highly correlated with treatment response [28]. The LASSO algorithm controls the number of selected variables by adjusting the parameter λ [29, 30]. To ensure selection of optimal radiomics, the SVM algorithm was used to calculate the radiomics score according to the selected parameters in the training cohort [24]. The parameters of the SVM method based on conventional radiomics were as follows: SVM-Type: eps-regression; SVM-Kernel: radial; Cost: 1; Gamma: 0.125; Epsilon: 0.1; Number of Support Vectors: 158. The parameters of the SVM method based on sub-regional radiomics were as follows: SVM-Type: eps-regression; SVM-Kernel: radial; Cost: 1; Gamma: 0.090; Epsilon: 0.1; Number of Support Vectors: 149. Therefore, the conventional radiomics model and SRRM were constructed and tested in the validation cohort.

### Statistical analysis

The performance of the SRRM was estimated in the training and validation cohorts. The optimal cutoff value for predicting response was defined using the Youden index and calculated using R software. We classified the samples into “SRRM-low” and “SRRM-high” groups based on the cut-off value. The Kaplan–Meier approach (log-rank test) was employed to analyze the PFS curves of the SRRM-low and SRRM-high groups, which were plotted with the *survminer* package in R software. The accuracies of different models were compared using the AUC and Akaike information criterion (AIC) in the *pROC* and *basicTrendline* packages in R software; higher AUC and lower AIC indicated a more accurate model predictive ability. Fisher’s exact test was used to analyze the frequency differences between both groups. Multiple comparison adjustments were made for Fisher’s exact test in the *fdrtool* package. Volcano plots were created using the *ggplot2* package in R software and were used to analyze the different frequencies in variant genes between the SRRM-low and SRRM-high groups. The statistical analyses for this study were performed using R version 3.5.1 (https://www.r-project.org/) and GraphPad Prism 7.01 (https://www.graphpad.com/). In addition, statistical significance was set at *P* < 0.05.

## Results

### Characteristics of patients

The basic clinical features of patients with NSCLC treated with ICIs in the training and validation cohorts are displayed in Table 1; there were 64 (40.25%) and 43 (40.95%) male patients, respectively. In the two cohorts, 100 (62.89%) and 71 (67.62%) patients, respectively, were > 60 years of age. The majority of individuals in the training (111 [69.81%]) and validation (81 [77.14%]) cohorts were “current smokers” or “ever smokers.” Our study revealed that in the training and validation cohorts, 47 (29.56%) and 27 (25.71%) patients, respectively, had a high TMB (≥ 10 muts/Mb). Furthermore, 15 (9.44%) and 10 (9.52%) individuals, respectively, had high PD-L1 expression (≥ 50%). In the training and validation cohorts, 38 (23.90%) and 23 (21.90%) patients, respectively, achieved clinical response.

**Table 1:** Characteristics of patients in the training and validation cohorts

Characteristic	Training cohort (*n* = 159)	Validation cohort (*n* = 105)
Sex		
Female	69 (43.39%)	49 (46.67%)
Male	64 (40.25%)	43 (40.95%)
NA	26 (16.36%)	13 (12.38%)
Age (years)		
≤ 60	33 (20.75%)	21 (20.00%)
> 60	100 (62.89%)	71 (67.62%)
NA	26 (16.36%)	13 (12.38%)
Smoking status		
Smoker	111 (69.81%)	81 (77.14%)
Non-smoker	22 (13.83%)	11 (10.48%)
NA	26 (16.36%)	13 (12.38%)
TMB		
High	47 (29.56%)	27 (25.71%)
Low	61 (38.36%)	47 (44.76%)
NA	51 (32.08%)	31 (29.53%)
PD-L1 expression		
High	15 (9.44%)	10 (9.53%)
Low	94 (59.11%)	63 (60.00%)
NA	50 (31.45%)	32 (30.47%)
Response status		
Response	38 (23.90%)	23 (21.91%)
Non-response	121 (76.10%)	82 (78.09%)

*NA* not available, *TMB* tumor mutational burden, *PD-L1* programmed death-ligand 1

### Selecting optimal sub-regional radiomic features and constructing the SRRM

**Fig. 2:**
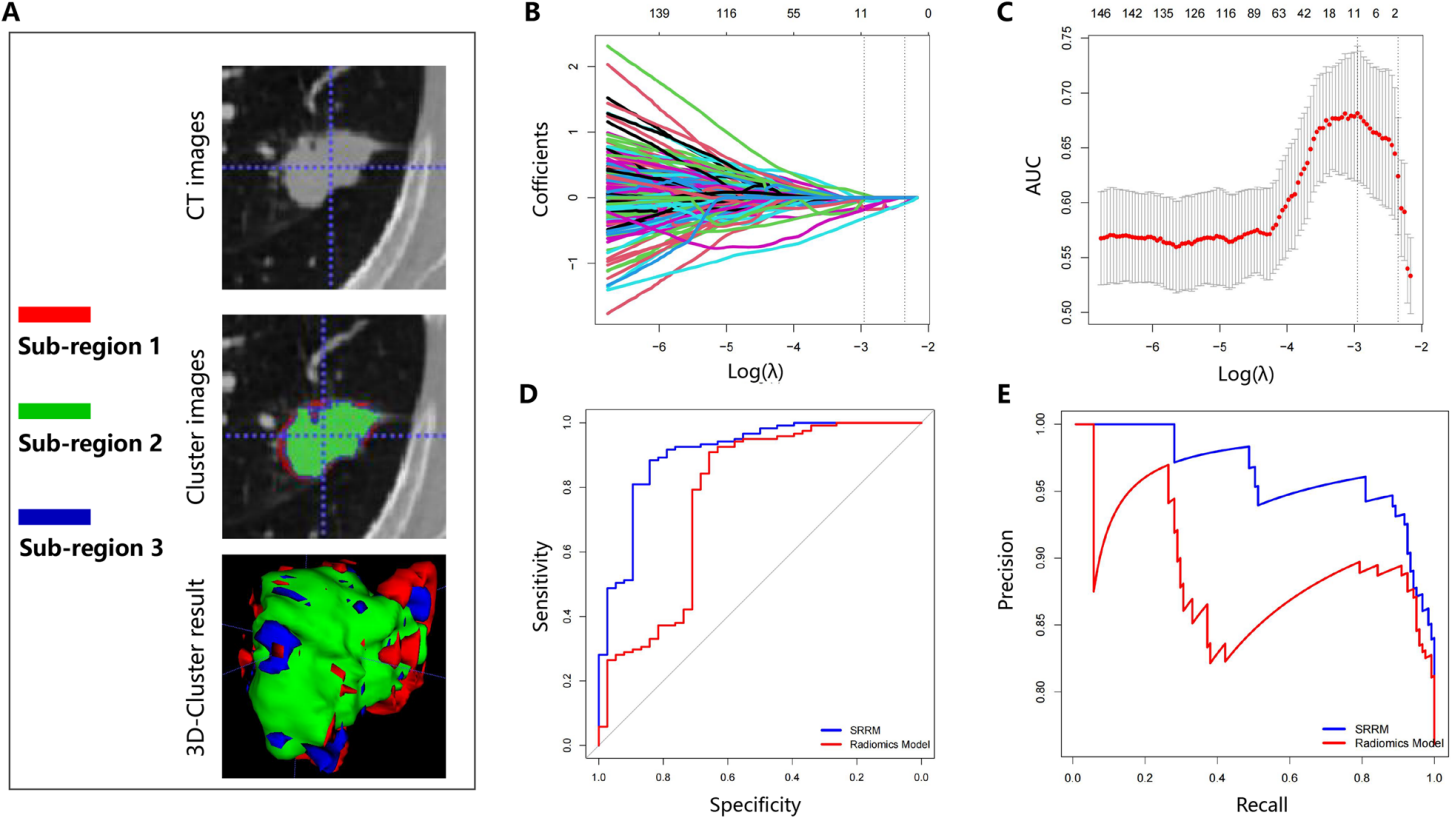
Least absolute shrinkage and selection operator was used to select features and construct the SRRM. **A** CT images, cluster images, and three-dimensional-cluster results are presented. **B**, **C** Optimal sub-regional radiomics (*n* = 11) were selected in patients with non-small cell lung cancer who underwent immunotherapy. **D** ROC curves for immunotherapy response prediction using the developed SRRM in the training cohort. **E** Precision–recall curves for response prediction using the developed SRRM in the training cohort. *SRRM* sub-regional radiomics model, *ROC* receiver operating characteristic, *CT* computed tomography, *AUC* area under the curve

The habitat images are presented in Fig. 2A, and the three colors represent different clusters. A total of 5688 radiomics features were extracted from three sub-regions. After eliminating redundant radiomic features, 1288 features remained for feature selection in each sub-region, resulting in a total of 3864 features. We used only 1288 features from the conventional radiomic features for further analysis. Based on the fivefold cross-validation, LASSO was applied to select optimal sub-regional radiomic features from the training cohort (Fig. 2B), and based on the analysis, 11 sub-regional radiomics features were eventually selected (Fig. 2C; Table 2). Similarly, in the training cohort, eight conventional radiomics were identified in patients with lung cancer who were treated with ICIs (Table 2). A total of 11 sub-regional radiomic features were employed to develop the SRRM to predict response to ICI treatment in the training cohort using SVM algorithms. Meanwhile, six conventional radiomics were used to develop the radiomics model in the training cohort. The SRRM demonstrated a higher AUC than the radiomics model in the training cohort (0.90 [95% confidence interval (CI) 0.84–0.96] and 0.77 [95% CI 0.67–0.87], respectively, both *P* < 0.001; Fig. 2D). The DeLong’s test for the two ROC curves was significant (*P* = 0.025). The recall and precision of the SRRM demonstrated a better performance than that of the radiomics model (recall: 87.42% vs. 76.72%, respectively; precision: 82.50% vs. 69.99%, respectively) (Fig. 2E). The AIC of the SRRM was significantly lower than that of the radiomics model (106.57 vs. 133.80, respectively).

**Table 2:** Selected radiomics associated with response in patients who received immunotherapy

Groups	Selected radiomics
Training cohort (sub-regional radiomics, *n* = 11)	*wavelet-LLL_glcm_InverseVariance* *wavelet-LHL_ngtdm_Busyness* *wavelet-HLH_glrlm_GrayLevelNonUniformityNormalized* *wavelet-HLH_glszm_GrayLevelNonUniformityNormalized* *wavelet-HHH_firstorder_Mean* *wavelet-HHH_firstorder_Kurtosis* *wavelet-LLH_ngtdm_Busyness* *wavelet-HLH_glszm_LargeAreaHighGrayLevelEmphasis* *squareroot_gldm_LargeDependenceLowGrayLevelEmphasis* *original_shape_Flatness* *wavelet-HHL_gldm_LargeDependenceEmphasis*
Training cohort (radiomics, *n* = 8)	*wavelet-LLL_glcm_MaximumProbability* *wavelet-LLL_firstorder_90Percentile* *wavelet-LHL_ngtdm_Busyness* *wavelet-HLL_glcm_MaximumProbability* *wavelet-HLH_gldm_DependenceEntropy* *wavelet-HLH_firstorder_Median* *squareroot_firstorder_Median* *squareroot_firstorder_90Percentile*

### SRRM testing in the validation cohort

**Fig. 3:**
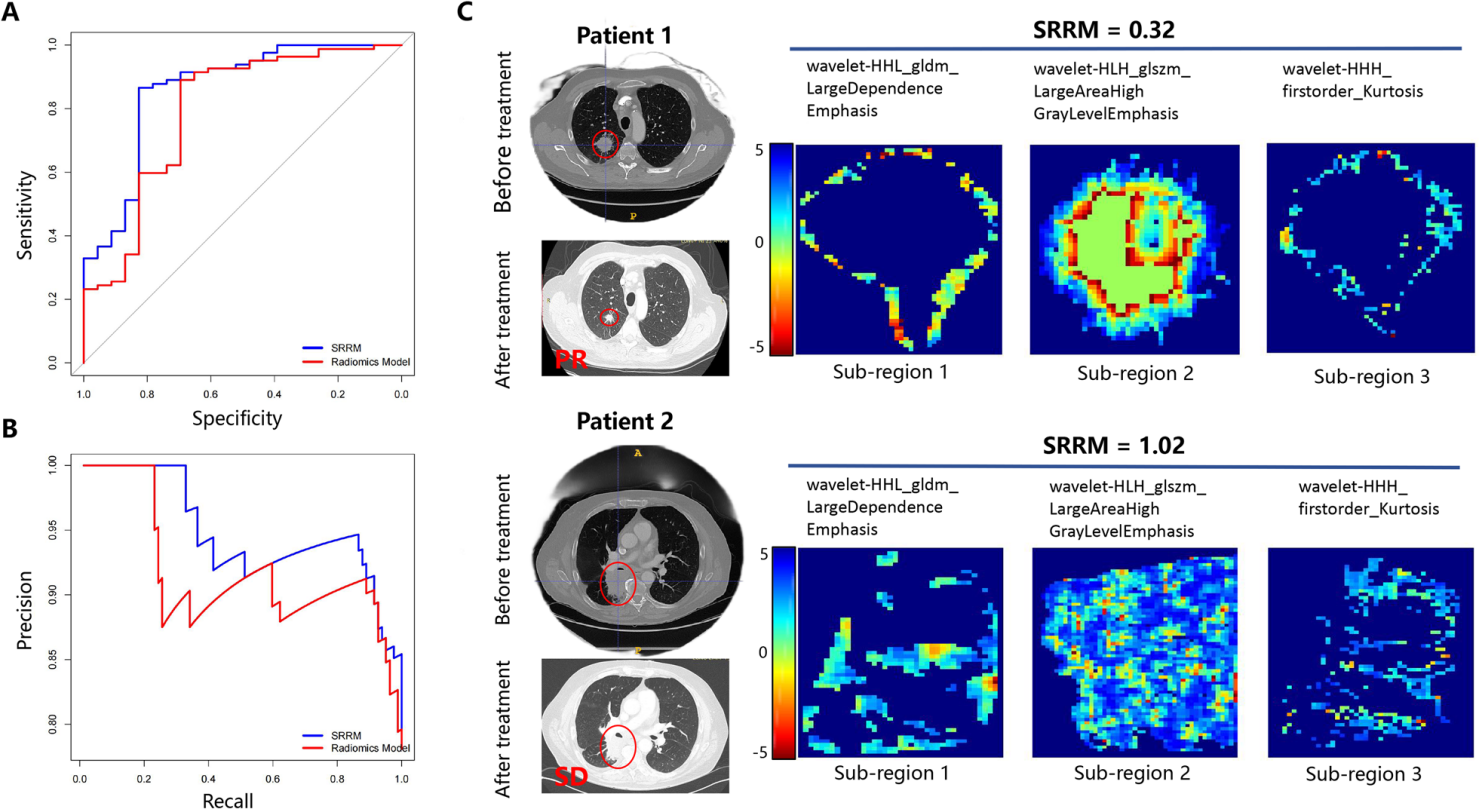
Validation of the SRRM for immunotherapy response prediction. **A** ROC curves for immunotherapy response prediction using the developed SRRM in the validation cohort. **B** Precision–recall curves for response prediction using the developed SRRM in the validation cohort. **C** Three sub-regional radiomics were visualized, as shown in the two patients. *SRRM* sub-regional radiomics model, *ROC* receiver operating characteristic

To analyze the performance of the models, the validation cohort was used for testing and the SRRM showed a higher AUC than the radiomics model (0.86 [95% CI 0.77–0.96] vs. 0.79 [95% CI 0.67–0.90], respectively, both *P* < 0.001; Fig. 3A). The DeLong’s test for two ROC curves was not significant (*P* = 0.377). The recall and precision of the SRRM demonstrated a better performance than that of the radiomics model (recall: 85.71% vs. 74.14%, respectively; precision: 79.00% vs. 70.13%, respectively) (Fig. 3B). Next, we further visualized the sub-regional radiomic features from the CT images of two patients. A good treatment response was observed in patient 1 according to the CT images and the SRRM value was significantly lower (0.32) than that of patient 2 (1.02) (Fig. 3C). The wavelet-HHL_gldm_Large Dependence Emphasis, wavelet-HLH_glszm_Large AreaHigh GrayLevel Emphasis, and wavelet-HHH_firstorder_Kurtosis features from the three different sub-regions were more concentrated in the tumor area in patient 1 than in patient 2, suggesting that the heterogeneity of the tumors may be different.

### Comparison of the SRRM with TMB score and PD-L1 expression

**Fig. 4:**
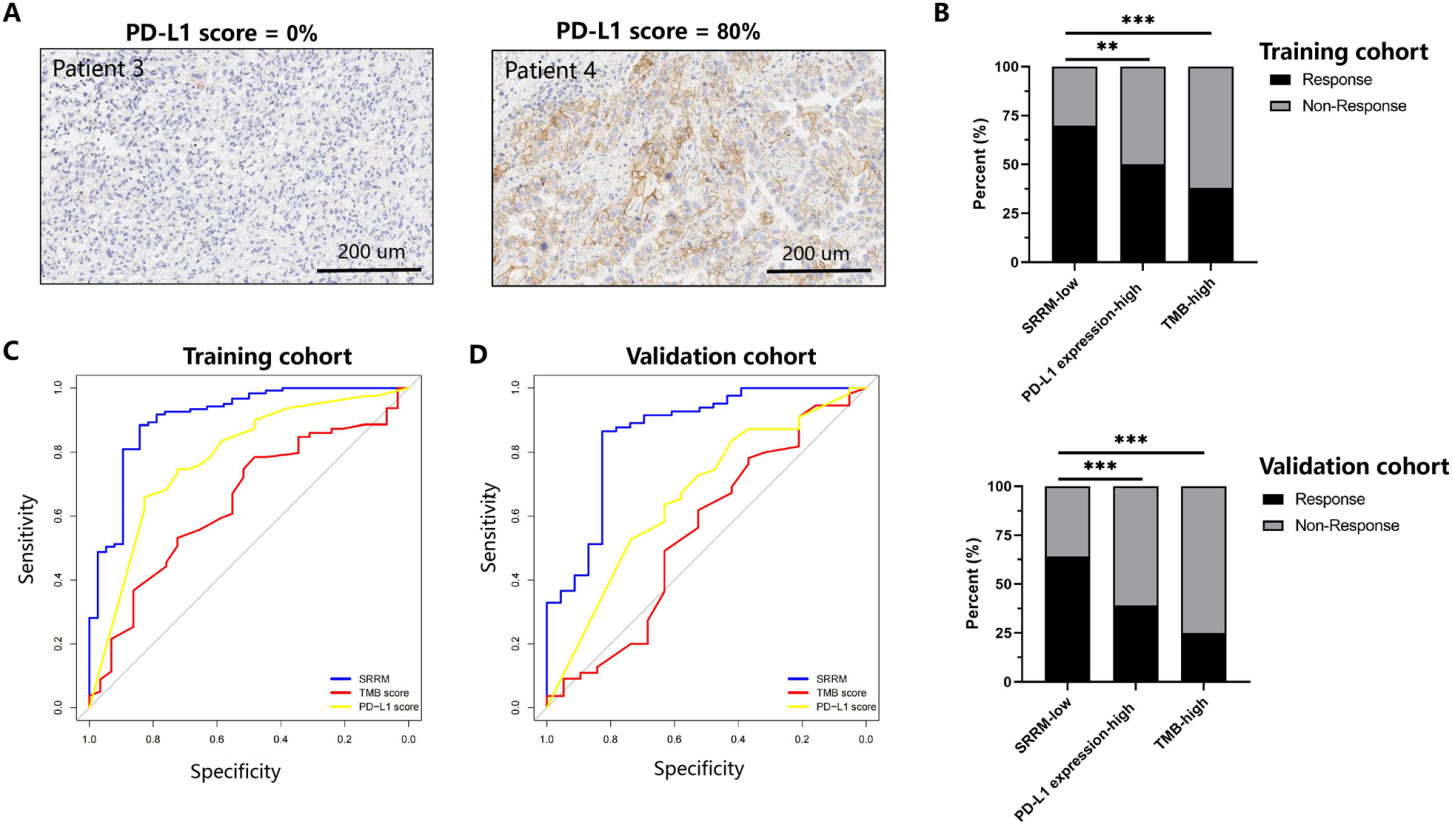
Comparison of the SRRM with TMB score and PD-L1 expression. **A** Images with negative (left) and positive (right) PD-L1 expression on immunohistochemistry are shown. **B** Different frequencies of immunotherapy among SRRM-low, PD-L1-high expression, and TMB-low groups. **C** ROC curves of predictive immunotherapy response using the SRRM, PD-L1 expression, and TMB score in the training and **D** validation cohorts. *PD-L1* programmed death-ligand 1, *TMB* tumor mutational burden, *SRRM* sub-regional radiomics model, *ROC* receiver operating characteristic

PD-L1 expression and TMB score were used as predictors of immunotherapy response, and it was relatively more challenging to compare them with the SRRM. Two samples from patient 3 (low PD-L1 expression) and patient 4 (high PD-L1 expression) from the validation cohort are presented in Fig. 4A. In the training cohort, the SRRM-low group (cutoff value < 0.936) had a higher treatment response than the PD-L1-high (70% vs. 50%, respectively, *P* = 0.003) and TMB-high groups (70% vs. 38%, respectively, *P <* 0.001) did (Fig. 4B). In the validation cohort, the SRRM-low group also had a higher treatment response than the PD-L1-high (64% vs. 39%, respectively, *P <* 0.001) and TMB-high groups (64% vs. 25%, respectively, *P <* 0.001) did (Fig. 4B). In the training cohort, the SRRM demonstrated a higher AUC (0.90 [95% CI 0.84–0.96], *P* < 0.001) than the PD-L1 expression (0.78 [95% CI 0.67–0.88], *P* < 0.001, DeLong’s test *P* = 0.032) and TMB score (0.64 [95% CI 0.52–0.75], *P* = 0.039, DeLong’s test *P* = 0.001) did (Fig. 4C). In the validation cohort, the SRRM demonstrated a higher AUC (0.86 [95% CI 0.77–0.96], *P* < 0.001) than the PD-L1 expression (0.66 [95% CI 0.51–0.80], *P* = 0.034, DeLong’s test *P* = 0.024) and TMB score (0.54 [95% CI 0.38–0.70], *P* = 0.552, DeLong’s test *P* = 0.001) did (Fig. 4D). Univariate analysis showed that TMB score, PD-L1 expression, and SRRM were associated with responses to immunotherapy (OR: 0.42 [95% CI 0.17–1.00], 0.15 [95% CI 0.05–0.37], and 27.18 [95% CI 9.16–95.87], *P* = 0.049, < 0.001, and < 0.001, respectively) (Table 3). Multivariate analysis revealed that PD-L1 expression and the SRRM were two independent predictors of the response to immunotherapy (OR: 0.09 [95% CI 0.02–0.33] and 37.32 [95% CI 10.00–196.64], *P* < 0.001 and < 0.001, respectively). Combination of the SRRM and PD-L1 expression showed high AUCs in the training and validation cohorts (0.90 [95% CI 0.83–0.97] and 0.88 [95% CI 0.81–0.96]; *P* < 0.001 and < 0.001, respectively).

**Table 3:** Univariate and multivariate analyses for the response in the training cohort

Variable	Univariate analysis		Multivariate analysis	
	OR (95% CI)	P-value	OR (95% CI)	P-value
Sex (female vs. male)	0.87 (0.36–2.05)	0.762	–	–
Age (years) (≤ 60 vs. > 60)	0.47 (0.12–1.40)	0.208	–	–
Smoking status (smoker vs. non-smoker)	0.49 (0.10–1.64)	0.293	–	–
TMB score (high vs. low)	0.42 (0.17–1.00)	0.049*	0.32 (0.07–1.14)	0.087
PD-L1 score (high vs. low)	0.15 (0.05–0.37)	< 0.001*	0.09 (0.02–0.33)	< 0.001*
SRRM (high vs. low)	27.18 (9.16–95.87)	< 0.001*	37.32 (10.00–196.64)	< 0.001*

*OR* odds ratio, *CI* confidence interval, *TMB* tumor mutational burden, *PD-L1* programmed death-ligand 1, *SRRM* sub-regional radiomics model

*P-value < 0.05

### PFS analysis of the SRRM in the training and validation cohorts

**Fig. 5:**
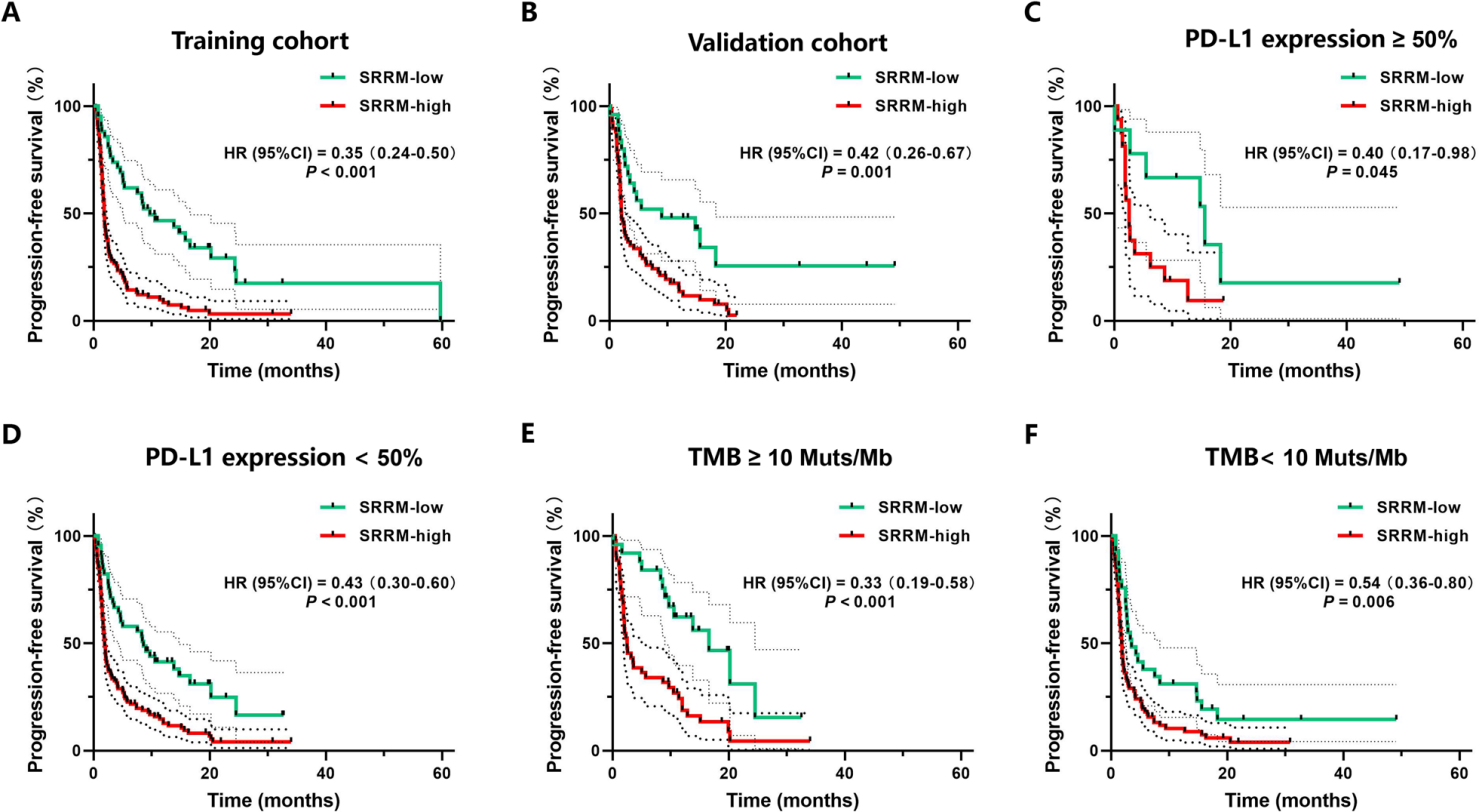
Association between the SRRM and PFS. **A** The SRRM predicts the PFS in the training and **B** validation cohorts. PFS curves of patients with **C** high PD-L1 expression (≥ 50%). **D** Low PD-L1 expression (< 50%). **E** High TMB score (≥ 10 muts/Mb), and **F** low TMB score (< 10 muts/Mb). *PFS* progression-free survival, *PD-L1* programmed death-ligand 1, *TMB* tumor mutational burden, *SRRM* sub-regional radiomics model, *CI* confidence interval, *HR* hazard ratio

To reveal the association between the SRRM and prognosis, we analyzed the PFS in the two cohorts. We found that the SRRM-low group had a longer median PFS than the SRRM-high group (9.70 vs. 1.80 months, respectively; hazard ratio (HR) = 0.35 [0.24–0.50], *P* < 0.001; Fig. 5A) in the training cohort. Similarly, the SRRM-low group had a longer median PFS than the SRRM-high group (9.00 vs. 2.10 months, respectively; HR = 0.42 [0.26–0.67], *P* = 0.001; Fig. 5B) in the validation cohort. The sub-group analysis was performed for PD-L1 expression and TMB score. In the PD-L1-high expression (≥ 50%) cohort, the SRRM-low group had a longer median PFS than the SRRM-high group (15.60 vs. 2.60 months, respectively; HR = 0.40 [0.17–0.98], *P* = 0.039; Fig. 5C). In the PD-L1-low expression (< 50%) cohort, the SRRM-low group had a longer median PFS than the SRRM-high group (8.60 vs. 1.90 months, respectively; HR = 0.43 [0.30–0.60], *P* < 0.001; Fig. 5D). Meanwhile, in the TMB-high (≥ 10 muts/Mb) cohort, the SRRM-low group had a longer median PFS than the SRRM-high group (16.60 vs. 2.50 months, respectively; HR = 0.33 [0.19–0.58], *P* < 0.001; Fig. 5E). Similarly, in the TMB-low (< 10 muts/Mb) cohort, the SRRM-low group had a longer median PFS than the SRRM-high group (3.50 vs. 1.80 months, respectively; HR = 0.54 [0.36–0.80], *P* = 0.006; Fig. 5F). Cox regression analysis showed that the SRRM, PD-L1 expression and TMB score were independent predictors of immunotherapy for NSCLC (*P* = 0.009, 0.016, and 0.018, respectively) in the combination of training and validation cohorts.

### Association between the SRRM and variant genes

**Fig. 6:**
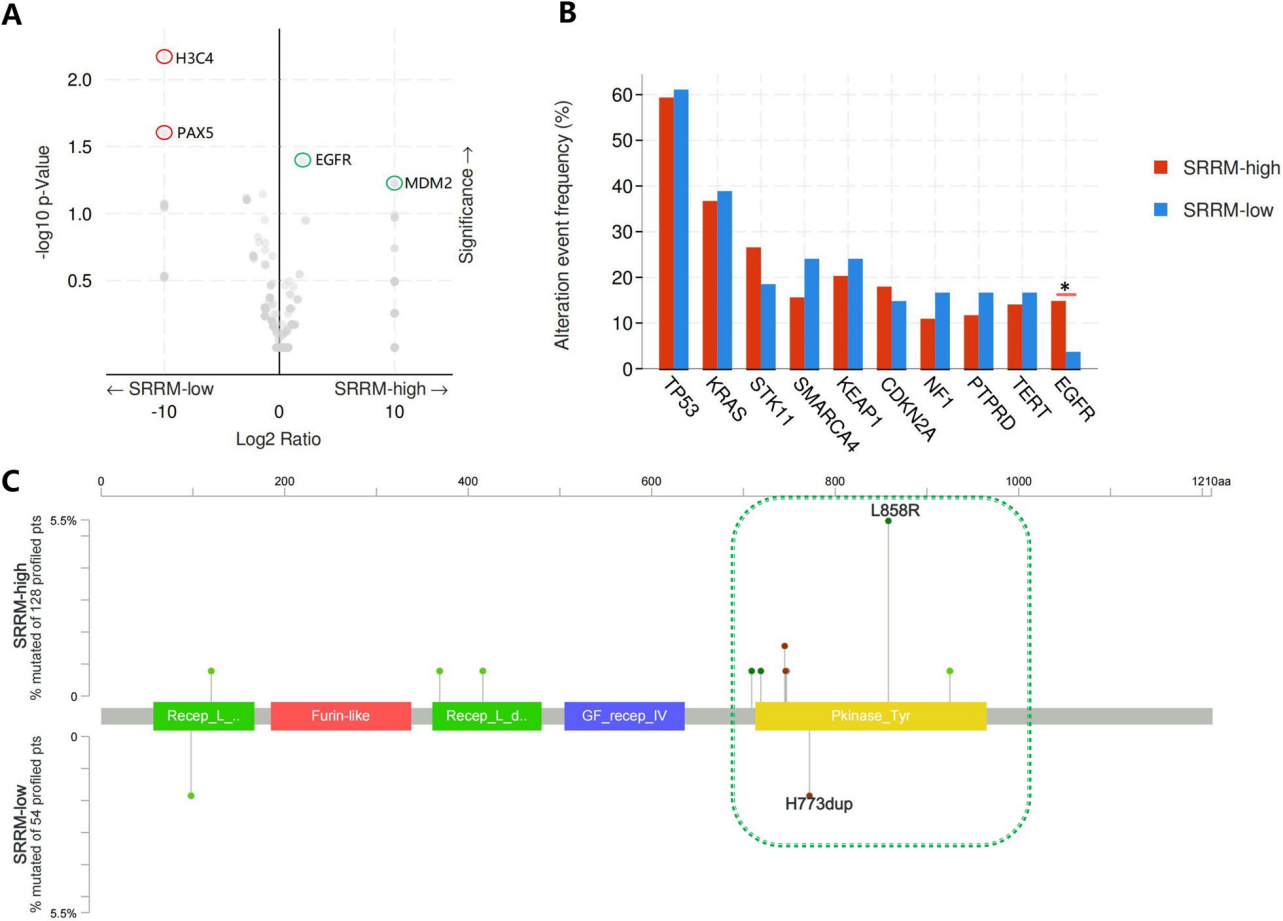
Relationship between the SRRM and variant genes. **A** Expressions of significantly enhanced variant genes in the SRRM-low and SRRM-high groups are shown using volcano plots. **B** Different frequencies of variant genes in the SRRM-low and SRRM-high groups. **C** Sites of *EGFR* mutation sub-types between the SRRM-low and SRRM-high groups are presented. *SRRM* sub-regional radiomics model

To analyze the association between SRRM and variant genes, different frequencies of genetic mutations, copy number variations, and fusion genes were compared. We found that *H3C4* (6p22.2) and *PAX5* (9p13.2) mutations were significantly enhanced in the SRRM-low group (*P* = 0.006 and 0.025, respectively) (Fig. 6A), whereas *EGFR* (7p11.2) mutation and *MDM2* amplification were significantly enhanced in the SRRM-high group (*P* = 0.040 and 0.059, respectively). The frequencies of the top 10 genetic mutations were compared between the SRRM-low and SRRM-high groups; only the frequency of *EGFR* mutation was significantly different between the two groups (Fig. 6B). We further explored the sub-mutation type of *EGFR* and found that exon 21 mutation (L858R, Missense_Mutation) was related to the SRRM-high group, whereas exon 20 mutation (H773dup, In_ Frame_Ins) was related to the SRRM-low group (Fig. 6C).

## Discussion

In this study, large sub-regional radiomics were extracted from pretreatment CT images in patients with NSCLC who received ICI treatment. The LASSO and SVM methods were used to select features and develop the SRRM. Our results showed that the SRRM had better performance in predicting immunotherapy response than conventional radiomics, TMB score, and PD-L1 expression. We also found that the SRRM-low group showed longer PFS than the SRRM-high group in the training and validation cohorts. The SRRM showed good PFS prediction ability of immunotherapy response regardless of the TMB score and PD-L1 expression status. Variant genes, including *H3C4* and *PAX5* mutations, were significantly associated with the SRRM-low group, whereas *EGFR* mutation was significantly associated with the SRRM-high group, especially the L858R mutation sub-type.

Identifying patients who can benefit from immunotherapy is a very active area of research; however, the current predictive biomarkers are relatively limited [31–33], and the search for new valuable markers is a worthwhile endeavor. Previous studies have used radiomics of the whole tumor and found that it can predict the response to immunotherapy to some extent [34, 35]; however, no study has focused on the correlation between the intratumoral heterogeneity and radiomics analysis of immunotherapy response. Recently, sub-regional radiomics has emerged as a novel approach, and the algorithm of mining tumor heterogeneity using sub-regional radiomics has attracted attention [36, 37]. The efficacy of concurrent chemoradiotherapy could be well predicted by sub-regional radiomics [38]. In this study, we used multi-center data to reveal the association between sub-regional radiomics and immunotherapy response, and found that the SRRM had better predictive performance and was superior to conventional radiomics. This may be attributed to the ability of sub-regional radiomics to better characterize tumor heterogeneity and better distinguish features between individual tumors, especially in the microenvironment. However, the intrinsic mechanism of this emerging sub-regional radiomics for immunotherapy prediction needs further analysis.

Several discoveries have been made in the field of immunotherapy biomarkers; however, only PD-L1 and TMB have been predominantly utilized in clinical practice [39, 40]. Although these biomarkers are relatively good, their prediction accuracy is still not ideal. It is suggested that both PD-L1 expression and TMB scores are based on tissue biopsies that sample only a small fraction of the tumor, and that the immunophenotypic and mutational features may differ between different regions of the tumor [41, 42]. Therefore, the findings of the PD-L1 and TMB biomarkers may be biased, affecting the prediction of immunotherapy response. CT imaging has a unique advantage in characterizing tumor panorama, which cannot be matched by any tumor histological sequencing. Although sub-regional radiomics is superior to conventional radiomics, it is not clear how it compares with the two classical biomarkers, TMB and PD-L1. In this study, we compared the SRRM with PD-L1 expression and TMB scores and found that the SRRM was indeed superior to PD-L1 expression and TMB score in predicting immunotherapy response. Meanwhile, the SRRM also performed well in predicting PFS regardless of PD-L1 expression or TMB score. It is also noteworthy that sub-regional radiomics is a non-invasive predictive method; comparatively easier to perform than PD-L1 and TMB analysis, which require tissue biopsy; less expensive; and more acceptable to patients than performing PD-L1 and TMB analysis. Consequently, herein, we proposed the development of new radiology biomarkers.

Comparison of genomic variants and radiomics is a valuable research topic; however, it has been underexplored. The relationship between sub-regional radiomics and genomic alterations in the context of immunotherapy for lung cancer has not been reported. We characterized the genes that were differentially mutated between the SRRM-low and SRRM-high groups using volcano maps and found that *H3C4* and *PAX5* mutations were associated with the immunotherapy-responsive SRRM-low group, which has not been reported previously. In addition, *EGFR* mutations and *MDM2* amplification were associated with the immunotherapy-resistant SRRM-high group, similar to previous reports [7, 43]. However, our study further showed that mutation of exon 21 of *EGFR* was associated with the SRRM-high group, whereas mutation of exon 20 was associated with the SRRM-low group. Different subtypes of *EGFR* mutations may have distinct impacts on the response to immunotherapy, which warrants further investigation.

This study had certain limitations. First, our study data were primarily derived from the MSKCC and TCIA cohorts, which represent an American population. Thus, the generalizability of the predictive power of these biomarkers in patients with lung cancer from different countries is uncertain, requiring further validation with data from more diverse clinical settings. Second, our study is retrospective, and we need to design multi-center studies to incorporate sub-regional radiomics in prospective cohorts. Finally, we did not have access to the overall survival (OS) data; therefore, we could only analyze the relationship between the SRRM and PFS. The correlation between OS and the SRRM needs to be evaluated in the future.

## Conclusions

We demonstrated that the SRRM based on pretreatment CT images was a novel and reliable model in predicting the response to ICI treatment in patients with NSCLC. The SRRM was also used to perform risk stratification of PFS, distinguishing between patients with rapid and slow progression. Our study provides new insights for predicting clinical outcomes of immunotherapy and could be useful for guiding clinical decision-making in the future.

## Data Availability

The data that support the findings of this study are available from the corresponding author on reasonable request.

## Abbreviations

AIC: Akaike information criterion
AUC: Area under the receiver operating characteristic curve
CT: Computed tomography
HR: Hazard ratio
ICIs: Immune checkpoint inhibitors
LASSO: Least absolute shrinkage and selection operator
MSKCC: Memorial Sloan Kettering Cancer Center
NGS: Next-generation sequencing
NSCLC: Non-small cell lung cancer
OS: Overall survival
PD-L1: Programmed death-ligand 1
PFS: Progression-free survival
ROC: Receiver operating characteristic
SRRM: Sub-regional radiomics model
SVM: Support vector machine
TMB: Tumor mutational burden
YI: Youden index
